# Molecular Phylogeny and Historical Biogeography of the Butterfly Tribe Aeromachini Tutt (*Lepidoptera: Hesperiidae*) from China

**DOI:** 10.3390/cells8040294

**Published:** 2019-03-29

**Authors:** Yuanyuan Li, Jianqing Zhu, Chen Ge, Ying Wang, Zimiao Zhao, Shuojia Ma, Ary A. Hoffmann, Nancy M. Endersby, Qunxiu Liu, Weidong Yu, Weibin Jiang

**Affiliations:** 1 College of Life Sciences, Shanghai Normal University, Shanghai 200234, China; liyuan9286@163.com (Y.L.); grechen9505@163.com (C.G.); wangyingsky2017@163.com (Y.W.); zhaozimiao818926@163.com (Z.Z.); mashuojia@126.com (S.M.); ywd@shnu.edu.cn (W.Y.); 2 Shanghai Zoological Park, Shanghai 200335, China; zzzjjq@gmail.com (J.Z.); liuqunxiu@126.com (Q.L.); 3 School of BioSciences, The University of Melbourne, Bio21 Institute, Parkville, VIC 3052, Australia; ary@unimelb.edu.au (A.A.H.); nancye@unimelb.edu.au (N.M.E.)

**Keywords:** Aeromachini, historical biogeography, phylogenetics, mitochondrial DNA

## Abstract

The butterfly tribe Aeromachini Tutt, 1906 is a large group of skippers. In this study, a total of 10 genera and 45 species of putative members of this tribe, which represent most of the generic diversity and nearly all the species diversity of the group in China, were sequenced for two mitochondrial genes and three nuclear genes (2093 bp). The combined dataset was analyzed with maximum likelihood inference using IQtree. We found strong support for monophyly of Aeromachini from China and support for the most recent accepted species in the tribe. Two paraphyletic genera within Aeromachini are presented and discussed. The divergence time estimates with BEAST and ancestral-area reconstructions with RASP provide a detailed description about the historical biogeography of the Aeromachini from China. The tribe very likely originated from the Hengduan Mountains in the late Ecocene and expanded to the Himalaya Mountains and Central China Regions. A dispersal-vicariance analysis suggests that dispersal events have played essential roles in the distribution of extant species, and geological and climatic changes have been important factors driving current distribution patterns.

## 1. Introduction

Butterflies have been exceptionally well studied as the subject of many taxonomic, ecological and evolutionary investigations [1]. However, the family Hesperiidae, commonly known as “skippers”, which includes around 4000 species, and has been largely ignored compared with systematic research on other butterfly families [2,3,4]. The first relatively comprehensive phylogeny of the family was inferred from three loci and morphological data for 196 genera by Warren et al. [5,6] only a decade ago and the result was mainly used to revise subfamily and tribal boundaries. Sahoo et al. [7] and Toussaint et al. [8] recently increased taxon and locus sampling and obtained a stronger support for most subfamilial and tribal level relationships. These results clarify a robust phylogenetic framework for Hesperiidae at higher taxonomic levels, especially based on anchored hybrid enrichment sequencing [8]. Despite this, these markers did not provide strong support to most complete generic and species-level phylogenetic relationships within the family because of the limited taxon sampling of a family that includes about 4000 species. The current study focuses on the tribe Aeromachini and constructs the phylogenetic relationships of the genera and species within the tribe to fill in one of the gaps at lower categories of Hesperiidae.

Aeromachini is a large and diverse tribe of Hesperiidae (subfamily Hesperiinae). Most species of Aeromachini are restricted geographically to the Oriental Region. Apart from the genus *Halpe*, most species of other genera of Aeromachini are distributed in the Sino-Himalayan Subregion [9]. The remaining species are found in the Palearctic Region and Afrotropical Region. The centers of species diversity are the eastern part of the Himalayan Region (Sikkim, the Kingdom of Bhutan, Assam of India and Tibetan southeastern area of China), north Myanmar and the Hengduan Mountains Region in southwest China. The common external features of the Aeromachini species are characterized by porrect palpi and forewing vein Cu_2_ (cubitus) arises opposite the origin of vein R_1_ (radius). Males of most species have a distortion at the end of the discal cell on hindwing. The length of discal cell is shorter than half length of hindwing. Most species of the tribe have a footstalk in the male genitalia. A pair of separated side panels is inserted in the juxta [10].

The earlier studies of Aeromachini are mainly focused on morphological classification, distribution and new species reports. [11,12,13]. Huang [10] constructed a phylogenetic relationship with 24 genera and 31 species of Hesperiidae based on 155 morphological characters in both external features and male genitalia, and defined the genera and species in the tribe Aeromachini in China. Despite this, due to the similar appearance and lack of effective and homologous morphological traits in most species of the family Hesperiidae [5,6,14,15,16], there are challenges in the traditional classification system and phylogenetic relationships based on morphological classification can be unclear. An example is the phylogenetic positions of *Aeromachus* and *Arnetta*, are uncertain in the study of Huang [10], because both these genera are not included in any group with robust support. 

To clarify the phylogenetic relationships of Aeromachini in China, we constructed phylogenetic trees with mitochondrial and nuclear genes, and compared these with the systematic relationships based on morphological characters. We used the mitochondrial genes Cytochrome Oxidase I (COI), Cytochrome Oxidase II (COII) and nuclear ribosomal DNA (rDNA) including the D3 region of 28S rRNA, and V4 and V7 regions of 18S rDNA. We sequenced a total of 122 specimens from 45 species, representing all major species groups. We constructed the phylogenetic trees under the methods Maximum likelihood (ML) using IQtree [17]. We also analyzed the origin of the tribe in China through prior studies on the distribution of Aeromachini species, and through estimating the times to the most recent common ancestor of the major lineages using relaxed-clock molecular dating. Huang et al. [18] recently reported a phylogenetic framework of Aeromachini species from East Asia, South Asia, Southeast Asia and the Far East, and inferred that the common ancestor of Aeromachini originated in Southeast Asia. In contrast to their wide sampling, we focused on the species from the most diversified region, South China. Our results help to understand the diversification and evolutionary history of Aeromachini in China and other groups with similar distribution patterns.

## 2. Materials and Methods

### 2.1. Taxon Sampling

We collected 122 specimens representing 45 species from 10 genera in the tribe Aeromachini in China (Table S1) across ten years. All specimens were caught in the field and preserved following dehydration in small envelopes. The preliminary species-level identification was based on traditional morphological characteristics of wings, genitalia, locality and additional information [10]. Three species in the same subfamily as Aeromachini and seven species from other subfamilies served as outgroups in the phylogenetic analyses. They are not a concern with the taxa chosen for the outgroup (Table S1).

### 2.2. DNA Extraction, PCR Amplification and Sequencing

The DNA was isolated from legs of adult butterflies using a QIAamp DNA Mini kit (Qiagen, Hilden, Germany) following the manufacturer’s instructions. The mitochondrial genes cytochrome oxidase subunit I (COI), cytochrome oxidase subunit II (COII) and three expansion segments (known as variable domains) of nuclear DNA (D3 region of 28S rDNA, V4 and V7 regions of 18S rDNA) were amplified using the primers described in Table 1 [19,20,21]. The PCR conditions were carried out following Jiang et al. [22]. Sequences were obtained by using an ABI 3730xl sequencer following the manufacturer’s recommendations. All novel sequences generated for this study were deposited in GenBank and accession numbers for these and other sequences were downloaded from GenBank (Table S1).

### 2.3. Phylogenetic Analyses

The sequence data of the mitochondrial dataset and nuclear dataset were aligned, translated to amino acid sequences to check for nuclear mitochondrial pseudogenes (numts), and pruned to remove redundant sequences with Bioedit v.7.0 [23]. MEGA v6.0 was used to calculate the genetic divergences based on the K2P model [24]. The best-fit partitioning scheme and corresponding nucleotide substitution models for the concatenated matrix were selected by PartitionFinder v2.1.1 [25] using the Bayesian Information Criterion (BIC). The GTR+R model is the best-fit substitution model for all four partitions. The concatenated supermatrix was analyzed with maximum likelihood (ML) inference using IQtree 1.4.2 [17]. To assess nodal support, we performed 1000 ultrafast bootstrap replicates and a SH-aLRT test with 1000 replicates. The UFBoot is largely unbiased compared to standard or alternative bootstrap strategies and SH-aLRT is conserved as standard bootstrap [26,27]. Only nodes with support values of UFBoot ≥ 80 and SH-aLRT ≥ 75 were considered robust.

### 2.4. Divergence Time Estimates 

The times to the most recent common ancestor (tmrca) of the major lineages were estimated using a relaxed-clock molecular dating estimation implemented in BEAST 1.5.2 [28]. Analyses using the HKY model of nucleotide substitution with gamma distributed rate variation among sites were performed and the Yule speciation method was assumed. We used the previously estimated age ranges (81–114 Mya) to calibrate the split between Hesperiidae and Hedylidae, the age ranges (35–55 Mya) between Hesperiinae and Heteropterinae [29,30], and a recently described fossil to constrain the minimum stem age of Hesperiinae (25 Mya) [31]. Chains were run for 50 million generations, with the first 20% discarded as burn-in. The results were summarized through TRACER 1.5 [32]. 

### 2.5. Ancestral Areas

**Figure 1:**
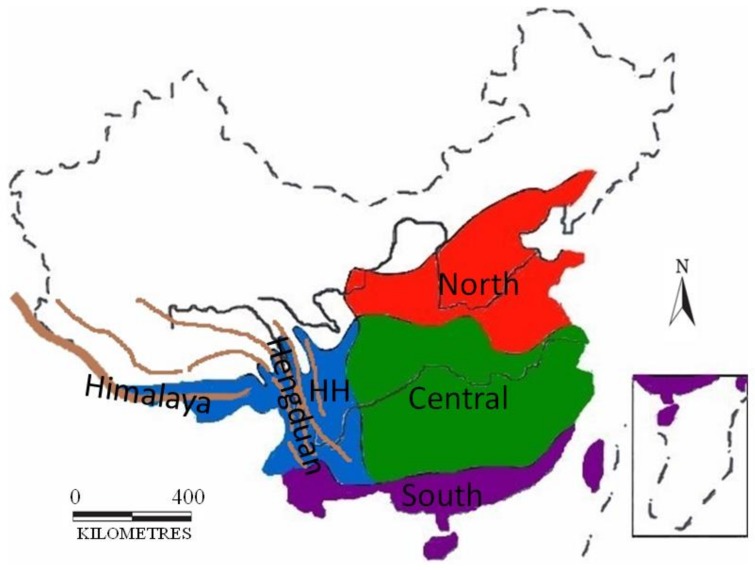
Four biogeographic units: HH, Himalaya-Hengduan Mountains Region; South, Southern China Region; Central, Central China Region; and North, Northern China Region.

To further discover the historical biogeography of Chinese Aeromachini, the distribution of all Aeromachini species in China (75) was summarized from the published studies and mapped [6,13,33,34,35,36,37,38,39,40,41,42]. Statistical dispersal-vicariance analysis (S-DIVA) and Bayesian binary MCMC (BBM) implemented in RASP 2.0 (Reconstruct Ancestral State in Phylogenies were used to infer the biogeographic history of the group [43,44]. Evolutionary events were inferred as dispersal, vicariance, extinction or standard speciation. Parameters for each analysis are presented in Table S2. The distributions of populations were divided into four biogeographic units based on the stochastic model of geographic range evolution [45,46] designated following Che et al. [47], Procheş and Ramdhani [48] and Zhang [49] as follows: (A) Himalaya-Hengduan Mountains Region; (B) Southern China Region; (C) Central China Region; and (D) Northern China Region (Figure 1). 

## 3. Results

### 3.1. Analysis and Tree Topology

**Figure 2:**
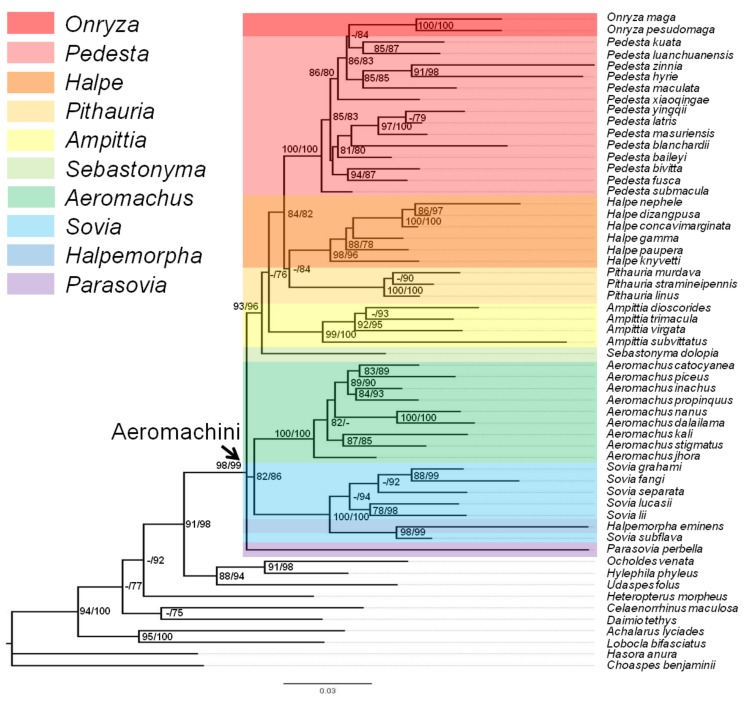
Maximum-likelihood phylogeny of Aeromachini sampled for this study. The phylogeny is inferred by IQTREE based on concatenated mitochondrial and nuclear genes (totaling 2084 bp). Numbers beside nodes are IQTREE ultrafast bootstrap and SH-aLRT values. The species from the ten genera are marked in different colors.

The information on number of nucleotide sites, variable sites, parsimony informative sites, fragment composition and accession numbers is listed in Table 2. Figure 2 shows the ML tree based on the dataset of the combined sequences and supports the monophyly of the tribe Aeromachini. In the phylogeny, there are three clades supported by high bootstrap values. Clade I contains most genera from the tribe: *Onryza*, *Pedesta*, *Halpe*, *Pithauria*, *Ampittia*, and *Sebastonyma*. Species belonging to the same genus form a monophyletic group except for the inclusion of *Onryza maga* and *Onryza pesudomaga* making *Pedesta* paraphyletic. Clade II contains three genera, *Aeromachus*, *Sovia* and *Halpemorpha*. *Aeromachus* is a monophyletic genus while *Sovia* is a paraphyletic group with the genus *Halpemorpha* forming a sister group to *Sovia subflava*. Clade III contains only *Parasovia*. Some closely related species with morphological separation are confirmed by our molecular data, e.g., the sympatric species *O. maga* and *O. pesudomaga* [42], *Pe. maculata* and *Pe. hyrie* [34], *Pe. latris* and *Pe. yingqii* [50], *So. lii* and *So. lucasii* [51], and *So. fangi* and *So. grahami* [36].

### 3.2. Divergence Time Estimates

**Figure 3:**
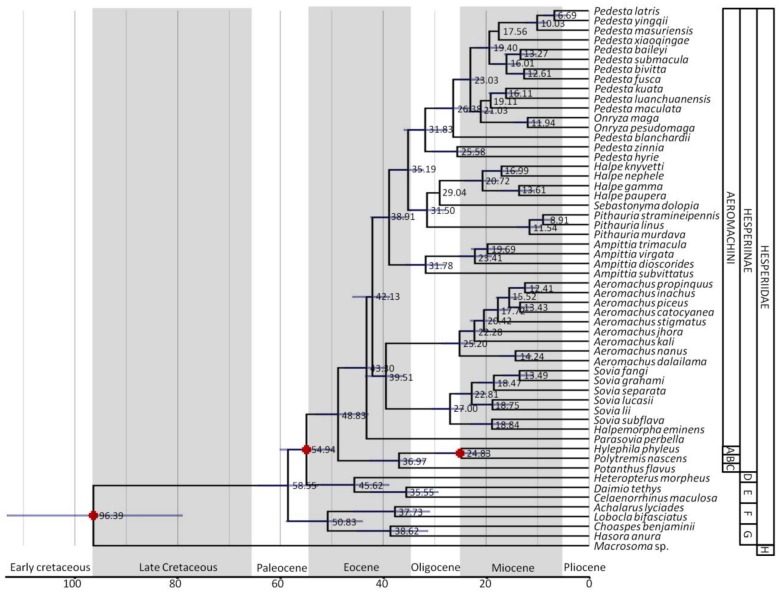
Chronogram of Aeromachini divergence based on mean tmrca estimates. The scale bar is in units of millions of years. Lettered nodes are those for which tmrca was estimated. A filled star denotes a node for which a prior calibration was used. A, Hesperiini; B, Baorini; C, Taractrocerini; D, Heteropterinae; E, Coeliadinae; F, Eudaminae, G, Pyrginae; H, Hedylidae.

The estimated divergence times for Aeromachini are presented in Figure 3. The initial divergences among Aeromachini were about 43 Mya (48–41, 95% HPD). Within Clade I, *Ampittia* diverged from other genera ≈39 Mya (42–36, 95% HPD). The split between *Onryza*-*Pedesta* and *Halpe*-*Pithauria*-*Sebastonyma* was dated at ≈35 Mya (39–32, 95% HPD). The diversification of *Pithauria* and *Halpe*-*Sebastonyma* occurred about 32 Mya (35–28, 95% HPD). *Halpe* diverged from *Sebastonyma* about 29 Mya. The diversification of Clade II occurred about 40 Mya (42–36, 95% HPD).

### 3.3. Ancestral Areas

**Figure 4:**
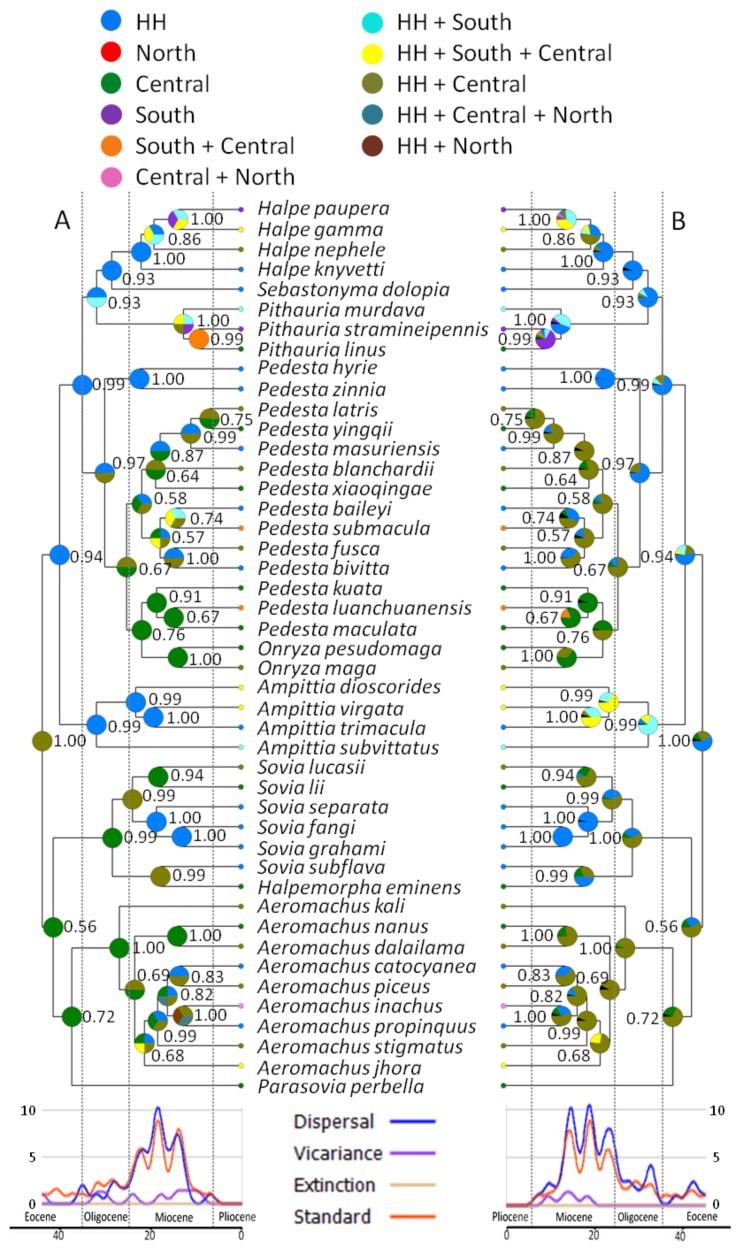
Biogeographic inference recovered with: (**A**) statistical dispersal-vicariance analysis (S-DIVA); and (**B**) Bayesian binary MCMC (BBM) in RASP 2.0. Pie charts represent the marginal probabilities for each alternative ancestral area: HH, Himalaya-Hengduan Mountains Region; South, Southern China Region; Central, Central China Region; and North, Northern China Region.

The most probable ancestral area and node frequency values from S-DIVA and BBM for major nodes are shown in Figure 4, and dispersal-vicariance-extinction plots juxtaposed with the phylogeny. Plots were similar in two models and dispersal was estimated to be dominant. For the S-DIVA, although some ambiguity and possible alternative resolutions exist, the highest likelihood estimates were consistent with the results of BBM. We considered it most likely for the hypotheses here.

The common ancestor of Aeromachini originated from the Hengduan Mountains, somewhere between the Himalaya-Hengduan Mountains Region and the Central China Region (Figure 4). A subsequent combination of vicariant and dispersal events separated two lineages of ancestral Aeromachini, giving rise to Clade I in the general area of the Himalaya-Hengduan Mountains Region and Clade II in the Central China Region. Within Clade I, an important dispersal event spread the clade from the Himalaya-Hengduan Mountains Region to Central China and, subsequently, there was a vicariant event within the genus *Pedesta*. The other dispersal event nearly simultaneously spread the *Halpe* + *Pithauria* + *Sebastonyma* lineage from the Himalaya-Hengduan Mountains Region to South and Central China. Within Clade II, the *Sovia* + *Halpemorpha* lineage diverged from the *Aeromachus* + *Parasovia* lineage in the Hengduan Mountains (between Himalaya-Hengduan Mountains Region and Central China Region) by a combination of vicariance and dispersal events. However, this result is contentious because node frequency is low (0.56). The common ancestor of *Sovia* and *Halpemorpha* occurred in the Central China Region and mainly spread back to the Himalaya-Hengduan Mountains Region. The common ancestor of *Aeromachus* and *Parasovia* also occurred in the Central China Region and became widespread.

## 4. Discussion

### 4.1. Taxonomic Implications

Well-defined taxonomic limits of Aeromachini have been a problem for many years. The members of Aeromachini were often classified in different tribes or generic groups by different entomologists [10]. Huang [10] could not verify the phylogenetic positions of *Aeromachus*, but did show the Areomachini from China consisted of at least nine genera based on morphological characters. We found strong support for the monophyly of Aeromachini from China and confirmed that *Aeromachus* belongs to this tribe based on molecular phylogenetic constructions in accord with Huang et al. [18]. Additionally, we collected samples from the most diversified region, South China, and made a further study based on their result.

We found two genera within Aeromachini are paraphyletic, *Pedesta* and *Sovia*. *Pedesta* includes all the traditionally-assigned *Pedesta* species and some *Thoressa* species that share similar morphological characters, and the genus is a sister genus of *Onryza* [18]. However, our results show that *Pedesta* is paraphyletic (Figure 2). The species from the genus *Onryza* and two species of *Pedesta*, *Pe*. *kuata* and *Pe*. *luanchuanensis*, firstly form a sister group relationship, and then cluster with the remaining *Pedesta* species to share a common ancestor. The species of *Onryza* share several characters with *Pedesta*: (a) forewings with white or yellow translucent spots; (b) gnathos developed and distally armed with small spines; and (c) uncus bifid or concave distally. Although we did not examine the type species of *Onryza* (*On. meiktila* distributed in Burma, Thailand and Laos) in this study, we believe *Pedesta* and *Onryza* should be synonymized based on the phylogenetic analyses and morphological characteristics. Based on the sample examined, we moved two Chinese *Onryza* species, *maga* and *pesudomaga*, to the genus *Pedesta*: *Pedesta maga* comb.nov. and *Pedesta pesudomaga* comb.nov.

The other paraphyletic genus is *Sovia* and the inclusion is *Halpemorpha eminens*. *Sovia* is rendered paraphyletic by the inclusion of *Halpemorpha eminens*. *Halpemorpha* was erected by Huang et al. [18] and includes only two species, *H. eminens* and *H. albipectus*. These two species were previously classified in *Sovia* by morphological criteria [12,52]. In this study, *eminens* and *So. subflava* are sister species with high support (Figure 2), and these cluster with the remaining species of *Sovia* form a monophyletic group. Although we included just one species of *Halpemorpha* in this study, we believe the genus should be re-examined.

The restructured genus *Ampittia*, which now includes *Ochus* based on the work of Huang et al. [18], is monophyletic with strong support in this study. Our results support Huang et al.’s [18] placement of *Ochus subvittatus* within *Ampittia*.

### 4.2. Historical Biogeography

Butterfly fossils are very rare and only 48 fossil butterfly species are named so far [31], making up a very low proportion of the total fossil record. The ages of butterflies and butterfly divergence time inferred in most previous studies are very different based on their calibrations on these few fossils [29,53,54,55,56]. The results of this study indicate that, although the first divergence time of the tribe Aeromachini (43 Mya) is earlier than the previously estimated times at 34 Mya, the ages of major evolutionary events in the taxa are consistent with previous studies [18]. The tribe Aeromachini probably originated in the late Ecocene and underwent adaptive radiation during the Oligocene and Miocene. These periods belong to the Tertiary, which is the heyday of the evolution of insect herbivores after a major extinction at the terminal Cretaceous (Cretaceous–Paleogene mass extinction, ~65 Mya) [55,57].

Huang et al. [18] reported that the common ancestor of Aeromachini originated in South China and Indochina. We further confirmed Aeromachini in China originated from the Hengduan Mountains. Most of the genera of Aeromachini originated 30–40 Mya between the Himalaya-Hengduan Mountains Region and the Central China Region, and began to diversify ca. 25 Mya (Figure 3). These estimates correspond well with the third of four major Qinghai–Tibetan Plateau uplifts believed to have occurred ca. 22–20, 15–13, 10–8, and 3.6–0 Mya [58,59,60,61], although the exact timings of these uplifts are still debated. Huang et al. [18] believed vicariance might play a significant role in the diversification of *Pedesta*. During the mid-Miocene, apart from the extensive uplift of the Himalaya-Hengduan Mountains Region, the dramatic crustal deformation induced by the Indo-Asian collision also contributed to the complicated landscapes in western China [62]. Massive mountains and deeply carved valleys acted as barriers to expansion and resulted in speciation.

Paleoecological records indicate that warm, humid and seasonal climates would have been common in southwestern China at least from the middle Miocene [63,64]. The mountains of southwestern China (Himalaya-Hengduan Mountains Region) can buffer regional climate variability and create stable climatic conditions [65]. Climatically stable refugia harbor not only greater concentrations of endemic species, but also high species diversity, and thus might be foci of speciation [66,67]. Combining the previous result of a dispersal-vicariance analysis, we propose that dispersal to adjacent areas has played essential roles within the Aeromachini, but speciation triggered by geological and climatic changes on the Qinghai–Tibetan Plateau also have been important factors in generating current biodiversity within this region.

## Figures and Tables

**Table 1:** Primer sequences and amplicon lengths of PCR products of target genes.

Gene/Region	Primers	Sequence (5′–3′)	Amplicon Length	Annealing Temperature	Reference
COI	HCO2198 LCO1490	TAAACTTCAGGGTGACCAAAAAATCA GGTCAACAAATCATAAAGATATTGG	487 bp	42 °C	[19]
COII	PIERRE EVA	AGAGCCTCTCCTTTAATAGAACA GAGACCATTACTTGCTTTCAGTCATCT	637 bp	45 °C	[20]
D3 region of 28S rDNA	CD3F CD3R	GGACCCGTCTTGAAACAC GCATAGTTCACCATCTTTC	240 bp	52 °C	[21]
V4 region of 18S rDNA	CV4F CV4R	TGGTGCCAGCAGCCGCGGTAA CCTCTAACGTCGCAATACGAATGCCC	381 bp	56 °C	[21]
V7 region of 18S rDNA	CV7F CV7R	CTTAAAGGAATTGACGGAGGGCACCACC GATTCCTTCAGTGTAGCGCGCGTG	400 bp	58 °C	[21]

**Table 2:** The information on gene fragment composition.

Gene Fragment	Nucleotide Sites	Variable Sites	Parsimony Informative Sites	A	T	C	G	Accession Numbers
COI	478	259	219	28.9%	39.5%	17.6%	13.9%	MK344780-MK344909
COII	625	293	260	35.6%	40.8%	13.5%	10.1%	MK344911-MK345027
D3 region of 28S rDNA	235	100	52	25.2%	19.3%	25.1%	30.4%	MK345289-MK345418
V4 region of 18S rDNA	392	69	37	24.3%	28.1%	20.2%	27.4%	MK345029-MK345156
V7 region of 18S rDNA	363	154	88	21.6%	24.4%	24.1%	29.8%	MK345158-MK345287

## Supplementary Materials

The following are available online at https://www.mdpi.com/2073-4409/8/4/294/s1, Figure S1: Diversity of Aeromachini (upperside and underside): (1) Aeromachus catocyanea; (2) Aeromachus dalailama; (3) Aeromachus inachus; (4) Aeromachus jhora; (5) Aeromachus kali; (6) Aeromachus nanus; (7) Aeromachus piceus; (8) Aeromachus propinquus; (9) Aeromachus stigmata; (10) Ampittia dioscorides; (11) Ampittia trimacula; (12) Ampittia virgata; (13) Halpe gamma; (14) Halpe knyvetti; (15) Halpe nephele; (16) Halpe pauper; (17) Ampittia subvittatus; (18) Onryza maga; (19) Onryza pesudomaga; (20) Parasovia perbella; (21) Pithauria linus; (22) Pithauria murdava; (23) Pithauria stramineipennis; (24) Sebastonyma dolopia; (25) Halpemorpha eminens; (26) Sovia fangi; (27) Sovia grahami; (28) Sovia lii; (29) Sovia lucasii; (30) Sovia separate; (31) Sovia subflava; (32) Pedesta baileyi; (33) Pedesta bivitta; (34) Pedesta blanchardii; (35) Pedesta fusca; (36) Pedesta hyrie; (37) Pededta kuata; (38) Pedesta latris; (39) Pedesta luanchuanensis; (40) Pedesta maculata; (41) Pedesta masuriensis; (42) Pedesta submacula; (43) Pedesta xiaoqingae; (44) Pedesta yingqii; (45) Pedesta zinnia. Scale bar = 10 mm, Table S1: Information on specimens used in this study, Table S2: Model parameters used in ancestral area estimation analysis.
