# Supplementary material for: Molecular Phylogeny and Historical Biogeography of the Butterfly Tribe Aeromachini Tutt (Lepidoptera: Hesperiidae) from China
Source: Cells. 2019 Mar 29;8(4):294. doi: 10.3390/cells8040294 (PMC6523876; doi:10.3390/cells8040294)
Supplement: Supplementary file 1 [file cells-08-00294-s001.pdf]

cells

MDPI

*Supplementary Material*

*Article*

# Molecular Phylogeny and Historical Biogeography of the Butterfly Tribe Aeromachini Tutt (Lepidoptera: Hesperiidae) from China

**Yuanyuan Li [1,†], Jianqing Zhu [2,†], Chen Ge [1], Ying Wang [1], Zimiao Zhao [1], Shuojia Ma [1], Ary A. Hoffmann [3], Nancy M. Endersby [3], Qunxiu Liu [2], Weidong Yu [1] and Weibin Jiang [1,*]**

[1] College of Life Sciences, Shanghai Normal University, Shanghai 200234, China; liyuan9286@163.com (Y.L.); gretchen9505@163.com (C.G.); wangyingsky2017@163.com (Y.W.); zhaozimiao818926@163.com (Z.Z.); mashuojia@126.com (S.M.); ywd@shnu.edu.cn (W.Y.)
[2] Shanghai Zoological Park, Shanghai 200335, China; zzzjjq@gmail.com (J.Z.); liuqunxiu@126.com (Q. L. )
[3] School of BioSciences, The University of Melbourne, Bio21 Institute, Parkville, Victoria 3052, Australia; ary@unimelb.edu.au (A.A.H.); nancye@unimelb.edu.au (N.M.E.)
* Correspondence: jiangwb@shnu.edu.cn; Tel.: +86 021 64321928
† These authors contributed equally to this work.

Received: 4 March 2019; Accepted: 23 March 2019; Published: 29 March 2019

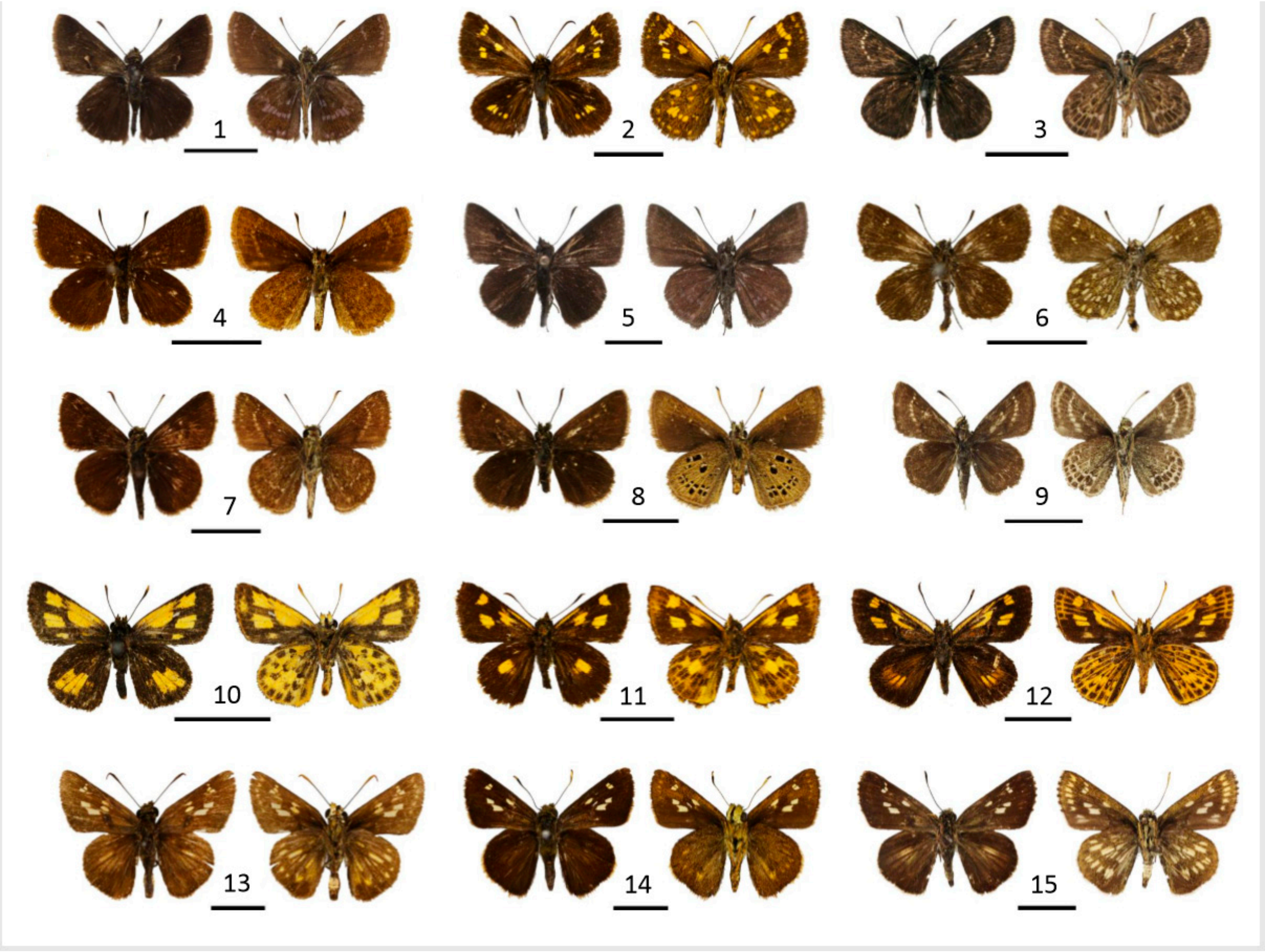
1
2
3
4
5
6
7
8
9
10
11
12
13
14
15

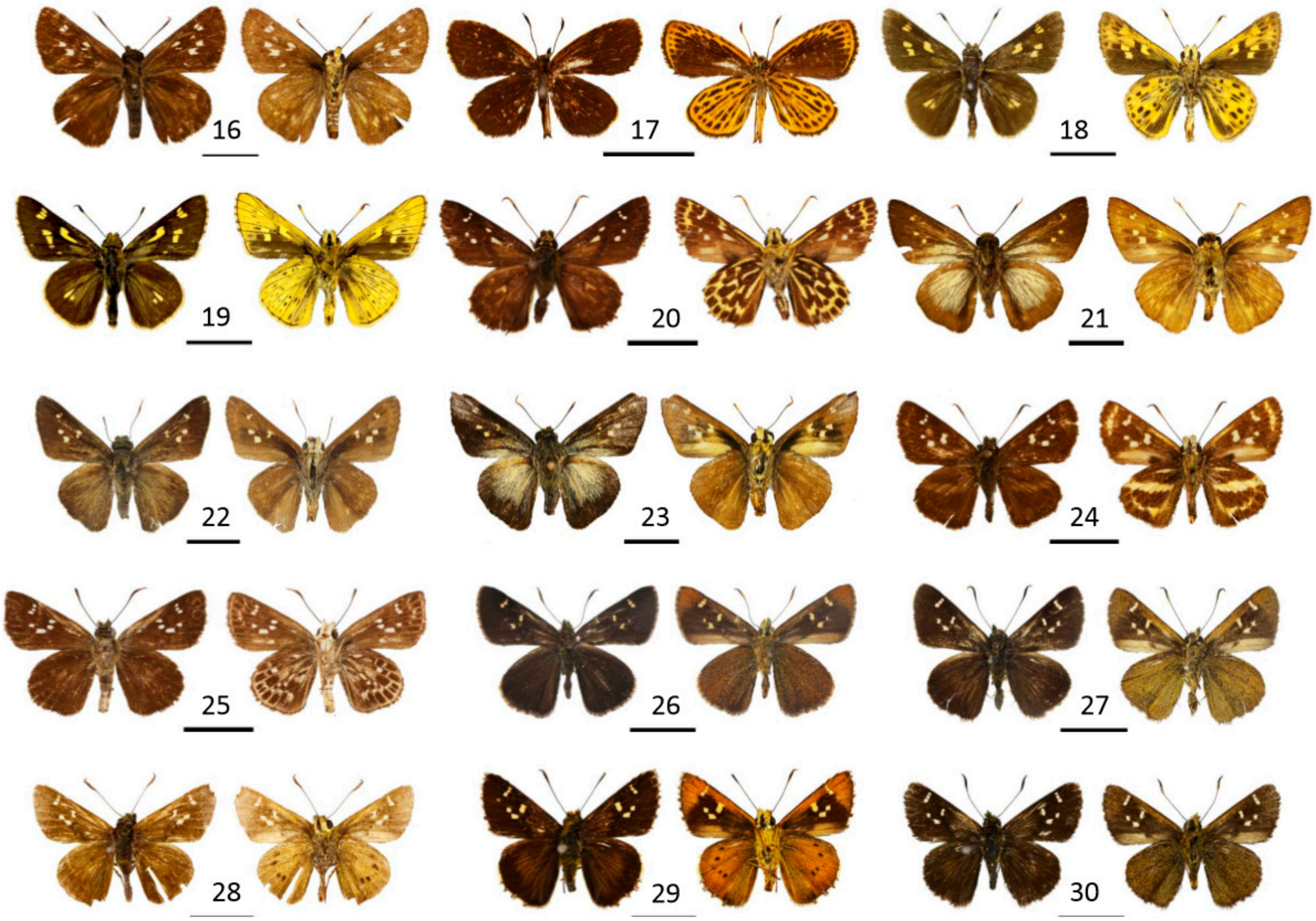
16
17
18
19
20
21
22
23
24
25
26
27
28
29
30

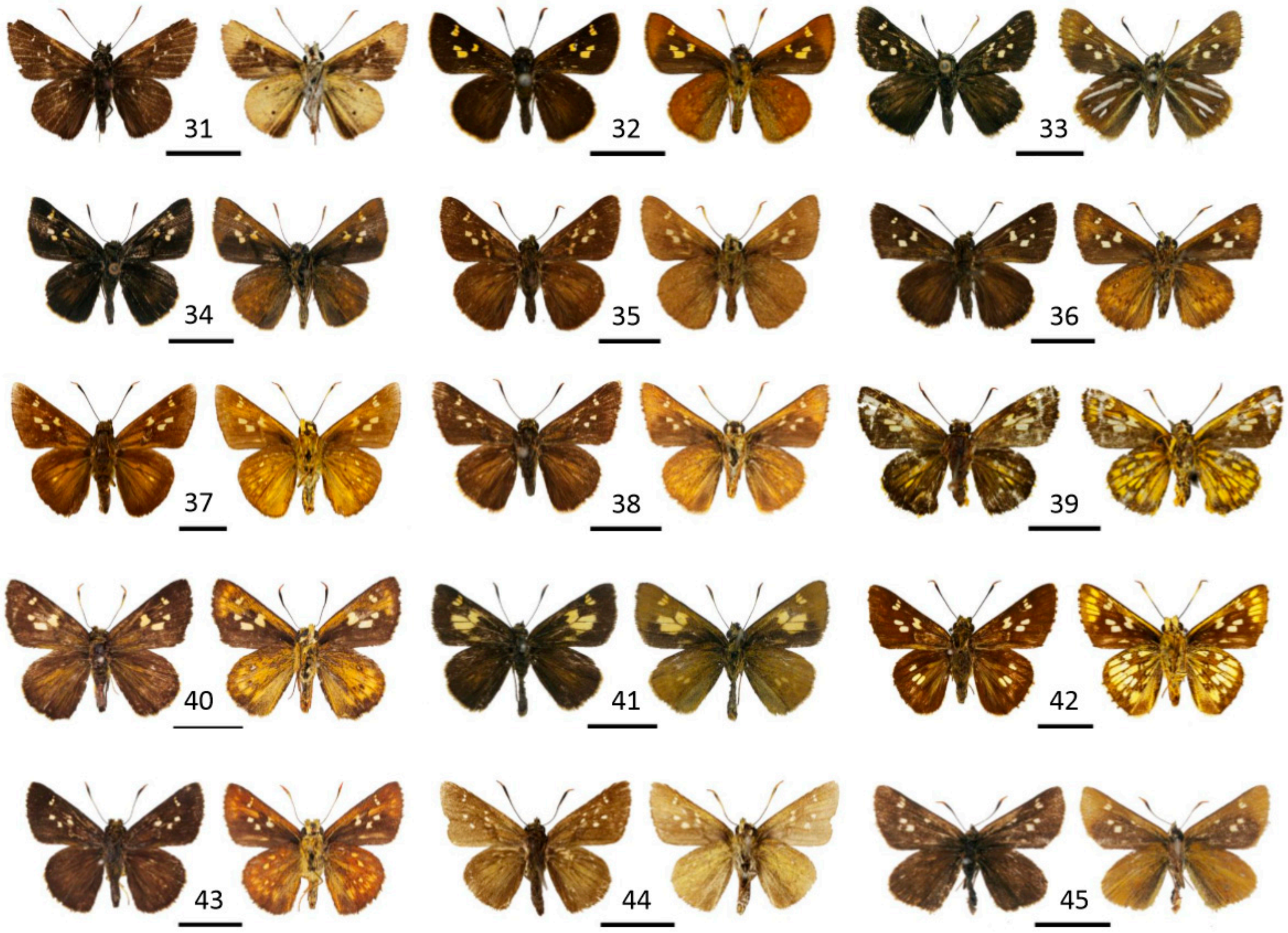


**Figure S1.** Diversity of Aeromachini (upperside and underside): (1) Aeromachus catocyanea; (2) Aeromachus dalailama; (3) Aeromachus inachus; (4) Aeromachus jhora; (5) Aeromachus kali; (6) Aeromachus nanus; (7) Aeromachus piceus; (8) Aeromachus propinquus; (9) Aeromachus stigmata; (10) Ampittia dioscorides; (11) Ampittia trimacula; (12) Ampittia virgata; (13) Halpe gamma; (14) Halpe knyvetti; (15) Halpe nephele; (16) Halpe pauper; (17) Ampittia subvittatus; (18) Onryza maga; (19) Onryza pesudomaga; (20) Parasovia perbella; (21) Pithauria linus; (22) Pithauria murdava;

(23) Pithauria stramineipennis; (24) Sebastonyma dolopia; (25) Halpemorpha eminens; (26) Sovia fangi; (27) Sovia grahami; (28) Sovia lii; (29) Sovia lucasii; (30) Sovia separate; (31) Sovia subflava; (32) Pedesta baileyi; (33) Pedesta bivitta; (34) Pedesta blanchardii; (35) Pedesta fusca; (36) Pedesta hyrie; (37) Pededta kuata; (38) Pedesta latris; (39) Pedesta luanchuanensis; (40) Pedesta maculata; (41) Pedesta masuriensis; (42) Pedesta submacula; (43) Pedesta xiaoqingae; (44) Pedesta yingqii; (45) Pedesta zinnia. Scale bar = 10 mm.

**Table S1.** Information on specimens used in this study.

| Species/Specimen ID | Sex | Locality | Date | Accession number | | | | |
|---|---|---|---|---|---|---|---|---|
| | | | | COI | COII | V4 | V7 | D3 |
| *Aeromachus catocyanea* 1 | ♂ | Shizong, Yunnan Province | 20150803 | MK344787 | MK344918 | MK345036 | MK345165 | MK345296 |
| *Aeromachus catocyanea* 2 | ♂ | Baoxing, Sichuan Province | 201507 | MK344788 | MK344919 | MK345037 | MK345166 | MK345297 |
| *Aeromachus dalailama* 1 | ♀ | Baoxing, Sichuan Province | 20150715 | MK344809 | MK344940 | MK345058 | MK345187 | MK345318 |
| *Aeromachus dalailama* 2 | ♂ | Baoxing, Sichuan Province | 20150720 | MK344810 | MK344941 | MK345059 | MK345188 | MK345319 |
| *Aeromachus dalailama* 3 | ♂ | Baoxing, Sichuan Province | 20150720 | MK344811 | MK344942 | MK345060 | MK345189 | MK345320 |
| *Aeromachus dalailama* 4 | ♂ | Linan, Zhejiang Province | 20140612 | MK344812 | MK344943 | MK345061 | MK345190 | MK345321 |
| *Aeromachus dalailama* 5 | ♀ | Linan, Zhejiang Province | 20140612 | MK344813 | MK344944 | MK345062 | MK345191 | MK345322 |
| *Aeromachus inachus* 1 | ♂ | Moganshan, Zhejiang Province | 20150928 | MK344789 | MK344920 | MK345038 | MK345167 | MK345298 |
| *Aeromachus inachus* 2 | ♂ | Moganshan, Zhejiang Province | 20150928 | MK344790 | MK344921 | MK345039 | MK345168 | MK345299 |
| *Aeromachus inachus* 3 | ♂ | Zhenjiang, Jiangsu Province | 20180601 | MK344791 | MK344922 | MK345040 | MK345169 | MK345300 |
| *Aeromachus jhora* 1 | ♀ | Mengla, Yunnan Province | 20160508 | MK344796 | MK344927 | MK345045 | MK345174 | MK345305 |
| *Aeromachus jhora* 2 | ♂ | Mangshi, Yunnan Province | 20150422 | MK344797 | MK344928 | MK345046 | MK345175 | MK345306 |
| *Aeromachus jhora* 3 | ♂ | Mengla, Yunnan Province | 20110104 | MK344798 | MK344929 | MK345047 | MK345176 | MK345307 |
| *Aeromachus jhora* 4 | ♀ | Mengla, Yunnan Province | 20130312 | MK344799 | MK344930 | MK345048 | MK345177 | MK345308 |
| *Aeromachus kali* 1 | ♂ | Jinxiu, Guangxi Province | 20110714 | MK344792 | MK344923 | MK345041 | MK345170 | MK345301 |
| *Aeromachus kali* 2 | ♂ | Gongshan, Yunnan Province | 20090526 | MK344793 | MK344924 | MK345042 | MK345171 | MK345302 |
| *Aeromachus nanus* 1 | ♂ | Tianmushan, Zhejiang Province | 20100529 | MK344806 | MK344937 | MK345055 | MK345184 | MK345315 |
| *Aeromachus nanus* 2 | ♀ | Tianmushan, Zhejiang Province | 20100601 | MK344807 | MK344938 | MK345056 | MK345185 | MK345316 |
| *Aeromachus nanus* 3 | ♂ | Tianmushan, Zhejiang Province | 20100529 | MK344808 | MK344939 | MK345057 | MK345186 | MK345317 |
| *Aeromachus piceus* 1 | ♂ | Baoxing, Sichuan Province | 201507 | MK344800 | MK344931 | MK345049 | MK345178 | MK345309 |
| *Aeromachus piceus* 2 | ♂ | Baoxing, Sichuan Province | 200907 | MK344801 | MK344932 | MK345050 | MK345179 | MK345310 |
| *Aeromachus piceus* 3 | ♂ | Baoxing, Sichuan Province | 200907 | MK344802 | MK344933 | MK345051 | MK345180 | MK345311 |
| *Aeromachus propinquus* 1 | ♂ | Kunming, Yunnan Province | 20130713 | MK344803 | MK344934 | MK345052 | MK345181 | MK345312 |
| *Aeromachus propinquus* 2 | ♂ | Shigu, Yunnan Province | 20160813 | MK344804 | MK344935 | MK345053 | MK345182 | MK345313 |
| *Aeromachus propinquus* 3 | ♂ | Kunming, Yunnan Province | 20130713 | MK344805 | MK344936 | MK345054 | MK345183 | MK345314 |
| *Aeromachus stigmatus* 1 | ♂ | Yingjiang, Yunnan Province | 20130518 | MK344794 | MK344925 | MK345043 | MK345172 | MK345303 |
| *Aeromachus stigmatus* 2 | ♂ | Chayu, Xizang Province | 20100829 | MK344795 | MK344926 | MK345044 | MK345173 | MK345304 |
| *Ampittia dioscorides*1 | ♂ | Songjiang, Shanghai | 20150713 | MK344780 | MK344911 | MK345029 | MK345158 | MK345289 |
| *Ampittia dioscorides*2 | ♀ | Zhenjiang, Jiangsu Province | 20180601 | MK344781 | MK344912 | MK345030 | MK345159 | MK345290 |
| *Ampittia dioscorides*3 | ♂ | Zhenjiang, Jiangsu Province | 20180601 | MK344782 | MK344913 | MK345031 | MK345160 | MK345291 |
| *Ampittia subvittatus* 1 | ♂ | Motuo, Xizang Autonomous Region | 20110805 | MK344879 | MK345001 | MK345126 | MK345257 | MK345388 |
| *Ampittia subvittatus* 2 | ♂ | Hekou, Yunnan Province | 20141005 | MK344880 | MK345002 | MK345127 | MK345258 | MK345389 |
| *Ampittia subvittatus* 3 | ♂ | Hekou, Yunnan Province | 20161022 | MK344881 | MK345003 | MK345128 | MK345259 | MK345390 |
| *Ampittia trimacula* 1 | ♂ | Baoxing, Sichuan Province | 200907 | MK344786 | MK344917 | MK345035 | MK345164 | MK345295 |
| *Ampittia virgata* 1 | ♂ | Wuyishan, Fujian Province | 20120512 | MK344783 | MK344914 | MK345032 | MK345161 | MK345292 |
| *Ampittia virgata* 2 | ♂ | Taishun, Zhejiang Province | 20120505 | MK344784 | MK344915 | MK345033 | MK345162 | MK345293 |
| *Ampittia virgata* 3 | ♂ | Wuyishan, Fujian Province | 20120512 | MK344785 | MK344916 | MK345034 | MK345163 | MK345294 |

| | | | | | | | | |
|---|---|---|---|---|---|---|---|---|
| *Halpe gamma* 1 | ♂ | Baoxing, Sichuan Province | 201507 | MK344825 | MK344956 | MK345074 | MK345203 | MK345334 |
| *Halpe gamma* 2 | ♀ | Taibei, Taiwan Province | 20110920 | MK344826 | MK344957 | MK345075 | MK345204 | MK345335 |
| *Halpe gamma* 3 | ♂ | Baoxing, Sichuan Province | 200906 | MK344827 | MK344958 | MK345076 | MK345205 | MK345336 |
| *Halpe knyvetti* 1 | ♂ | Motuo, Xizang Autonomous Region | 20140728 | MK344838 | — | — | MK345216 | MK345347 |
| *Halpe knyvetti* 2 | ♂ | Motuo, Xizang Autonomous Region | 20130725 | MK344839 | — | — | MK345217 | MK345348 |
| *Halpe nephele* 1 | ♂ | Baoxing, Sichuan Province | 200906 | MK344828 | MK344959 | MK345077 | MK345206 | MK345337 |
| *Halpe nephele* 2 | ♂ | Baoxing, Sichuan Province | 200906 | MK344829 | MK344960 | MK345078 | MK345207 | MK345338 |
| *Halpe paupera* 1 | ♀ | Lingui, Guangxi Province | 20110713 | MK344824 | MK344955 | MK345073 | MK345202 | MK345333 |
| *Halpemorpha eminens* 1 | ♂ | Nanling, Guangdong Province | 20100710 | MK344892 | — | MK345139 | MK345270 | MK345401 |
| *Onryza maga* 1 | ♂ | Maoershan, Guangxi Province | 20120725 | MK344814 | MK344945 | MK345063 | MK345192 | MK345323 |
| *Onryza maga* 2 | ♀ | Wuyishan, Fujian Province | 20160830 | MK344815 | MK344946 | MK345064 | MK345193 | MK345324 |
| *Onryza maga* 3 | ♂ | Maoershan, Guangxi Province | 20120725 | MK344816 | MK344947 | MK345065 | MK345194 | MK345325 |
| *Onryza maga* 4 | ♀ | Tianmushan, Zhejiang Province | 20110628 | MK344817 | MK344948 | MK345066 | MK345195 | MK345326 |
| *Onryza maga* 5 | ♀ | Tianmushan, Zhejiang Province | 20110628 | MK344818 | MK344949 | MK345067 | MK345196 | MK345327 |
| *Onryza maga* 6 | ♀ | Hualian, Taiwan Province | 20070704 | MK344819 | MK344950 | MK345068 | MK345197 | MK345328 |
| *Onryza maga* 7 | ♀ | Hualian, Taiwan Province | 20070705 | MK344820 | MK344951 | MK345069 | MK345198 | MK345329 |
| *Onryza pesudomaga* 1 | ♂ | Linan, Zhejiang Province | 20140613 | MK344821 | MK344952 | MK345070 | MK345199 | MK345330 |
| *Onryza pesudomaga* 2 | ♂ | Qingliangfeng, Zhejiang Province | 20140614 | MK344822 | MK344953 | MK345071 | MK345200 | MK345331 |
| *Onryza pesudomaga* 3 | ♂ | Qingliangfeng, Zhejiang Province | 20140614 | MK344823 | MK344954 | MK345072 | MK345201 | MK345332 |
| *Parasovia perbella* 1 | ♂ | Nanling, Guangdong Province | 20100612 | MK344878 | — | MK345125 | MK345256 | MK345387 |
| *Pedesta baileyi* 1 | ♂ | Tengchong, Yunnan Province | 20130426 | MK344852 | MK344979 | MK345099 | MK345230 | MK345361 |
| *Pedesta baileyi* 2 | ♀ | Motuo, Xizang Autonomous Region | 20130811 | MK344853 | MK344980 | MK345100 | MK345231 | MK345362 |
| *Pedesta bivitta* 1 | ♂ | Yulong, Yunnan Province | 20140531 | MK344849 | MK344976 | MK345096 | MK345227 | MK345358 |
| *Pedesta bivitta* 2 | ♂ | Yulong, Yunnan Province | 20140614 | MK344850 | MK344977 | MK345097 | MK345228 | MK345359 |
| *Pedesta bivitta* 3 | ♂ | Yulong, Yunnan Province | 20140614 | MK344851 | MK344978 | MK345098 | MK345229 | MK345360 |
| *Pedesta blanchardii* 1 | ♂ | Baoxing, Sichuan Province | 201507 | MK344856 | MK344983 | MK345103 | MK345234 | MK345365 |
| *Pedesta blanchardii* 2 | ♂ | Erlangshan, Sichuan Province | 20120713 | MK344857 | MK344984 | MK345104 | MK345235 | MK345366 |
| *Pedesta fusca* 1 | ♂ | Nanling, Guangdong Province | 20140615 | MK344873 | MK344996 | MK345120 | MK345251 | MK345382 |
| *Pedesta fusca* 2 | ♂ | Nanling, Guangdong Province | 20130704 | MK344874 | MK344997 | MK345121 | MK345252 | MK345383 |
| *Pedesta fusca* 3 | ♂ | Tengchong, Yunnan Province | 20160808 | MK344875 | MK344998 | MK345122 | MK345253 | MK345384 |
| *Pedesta fusca* 4 | ♂ | Ningshan, Shanxi Province | 20160610 | MK344876 | MK344999 | MK345123 | MK345254 | MK345385 |
| *Pedesta fusca* 5 | ♂ | Kang County, Gansu Province | 20160608 | MK344877 | MK345000 | MK345124 | MK345255 | MK345386 |
| *Pedesta hyrie* 1 | ♂ | Motuo, Xizang Autonomous Region | 20130813 | MK344871 | — | MK345118 | MK345249 | MK345380 |
| *Pedesta kuata* 1 | ♂ | Gutianshan, Zhejiang Province | 20130617 | MK344865 | MK344990 | MK345112 | MK345243 | MK345374 |
| *Pedesta kuata* 2 | ♂ | Qingliangfeng, Zhejiang Province | 20120709 | MK344866 | MK344991 | MK345113 | MK345244 | MK345375 |
| *Pedesta kuata* 3 | ♂ | Gutianshan, Zhejiang Province | 20130617 | MK344867 | MK344992 | MK345114 | MK345245 | MK345376 |
| *Pedesta kuata* 4 | ♂ | Tianmushan, Zhejiang Province | 20100617 | MK344868 | MK344993 | MK345115 | MK345246 | MK345377 |
| *Pedesta kuata* 5 | ♂ | Qingliangfeng, Zhejiang Province | 20120709 | MK344869 | MK344994 | MK345116 | MK345247 | MK345378 |
| *Pedesta latris* 1 | ♂ | Kunming, Yunnan Province | 20150603 | MK344859 | MK344985 | MK345106 | MK345237 | MK345368 |
| *Pedesta latris* 2 | ♂ | Yanling, Hunan Province | 20160613 | MK344860 | MK344986 | MK345107 | MK345238 | MK345369 |
| *Pedesta latris* 3 | ♂ | Hutiaoxia, Yunnan Province | 20140602 | MK344861 | MK344987 | MK345108 | MK345239 | MK345370 |
| *Pedesta latris* 4 | ♂ | Hutiaoxia, Yunnan Province | 20140602 | MK344862 | MK344988 | MK345109 | MK345240 | MK345371 |
| *Pedesta latris* 5 | ♂ | Baoxing, Sichuan Province | 201506 | MK344863 | MK344989 | MK345110 | MK345241 | MK345372 |

| | | | | | | | | |
|---|---|---|---|---|---|---|---|---|
| *Pedesta luanchuanensis* 1 | ♂ | Taishun, Zhejiang Province | 20180616 | MK344872 | MK344995 | MK345119 | MK345250 | MK345381 |
| *Pedesta maculata* 1 | ♂ | Jinxiu, Guangxi Province | 20110708 | MK344847 | — | MK345094 | MK345225 | MK345356 |
| *Pedesta maculata* 2 | ♂ | Jinxiu, Guangxi Province | 20110723 | MK344848 | — | MK345095 | MK345226 | MK345357 |
| *Pedesta masuriensis* 1 | ♂ | Gongshan, Yunnan Province | 20090607 | MK344854 | MK344981 | MK345101 | MK345232 | MK345363 |
| *Pedesta masuriensis* 2 | ♂ | Gongshan, Yunnan Province | 20090526 | MK344855 | MK344982 | MK345102 | MK345233 | MK345364 |
| *Pedesta submacula* 1 | ♀ | Wuyishan, Fujian Province | 20120512 | MK344840 | MK344969 | MK345087 | MK345218 | MK345349 |
| *Pedesta submacula* 2 | ♀ | Tianmushan, Zhejiang Province | 20130810 | MK344841 | MK344970 | MK345088 | MK345219 | MK345350 |
| *Pedesta submacula* 3 | ♂ | Wuyishan, Fujian Province | 20120512 | MK344842 | MK344971 | MK345089 | MK345220 | MK345351 |
| *Pedesta submacula* 4 | ♂ | Tianmushan, Zhejiang Province | 20130810 | MK344843 | MK344972 | MK345090 | MK345221 | MK345352 |
| *Pedesta submacula* 5 | ♂ | Tianmushan, Zhejiang Province | 20100617 | MK344844 | MK344973 | MK345091 | MK345222 | MK345353 |
| *Pedesta submacula* 6 | ♀ | Tianmushan, Zhejiang Province | 20100603 | MK344845 | MK344974 | MK345092 | MK345223 | MK345354 |
| *Pedesta submacula* 7 | ♂ | Baoji, Shanxi Province | 20100727 | MK344846 | MK344975 | MK345093 | MK345224 | MK345355 |
| *Pedesta xiaoqingae* 1 | ♂ | Jinxiu, Guangxi Province | 20110713 | MK344864 | — | MK345111 | MK345242 | MK345373 |
| *Pedesta yingqii* 1 | ♂ | Baoji, Shanxi Province | 20090603 | MK344858 | — | MK345105 | MK345236 | MK345367 |
| *Pedesta zinnia* 1 | ♀ | Xianggelila, Yunnan Province | 20090614 | MK344870 | — | MK345117 | MK345248 | MK345379 |
| *Pithauria linus* 1 | ♂ | Baoxing, Sichuan Province | 201507 | MK344884 | MK345006 | MK345131 | MK345262 | MK345393 |
| *Pithauria linus* 2 | ♂ | Lushan, Sichuan Province | 20150718 | MK344885 | MK345007 | MK345132 | MK345263 | MK345394 |
| *Pithauria linus* 3 | ♂ | Jinxiu, Guangxi Province | 20110729 | MK344886 | MK345008 | MK345133 | MK345264 | MK345395 |
| *Pithauria murdava* 1 | ♂ | Yuanjiang, Yunnan Province | 20100907 | MK344882 | MK345004 | MK345129 | MK345260 | MK345391 |
| *Pithauria murdava* 2 | ♂ | Yuanjiang, Yunnan Province | 20100710 | MK344883 | MK345005 | MK345130 | MK345261 | MK345392 |
| *Pithauria stramineipennis* 1 | ♂ | Jinghong, Yunnan Province | 20170930 | MK344887 | MK345009 | MK345134 | MK345265 | MK345396 |
| *Pithauria stramineipennis* 2 | ♂ | Jinghong, Yunnan Province | 20170930 | MK344888 | MK345010 | MK345135 | MK345266 | MK345397 |
| *Sebastonyma dolopia* 1 | ♂ | Dulongjiang, Yunnan Province | 20150723 | MK344889 | MK345011 | MK345136 | MK345267 | MK345398 |
| *Sebastonyma dolopia* 2 | ♂ | Motuo, Xizang Autonomous Region | 20140724 | MK344890 | MK345012 | MK345137 | MK345268 | MK345399 |
| *Sebastonyma dolopia* 3 | ♂ | Dulongjiang, Yunnan Province | 20150723 | MK344891 | MK345013 | MK345138 | MK345269 | MK345400 |
| *Sovia fangi* 1 | ♂ | Weixi, Yunnan Province | 20100709 | MK344896 | MK345015 | MK345143 | MK345274 | MK345405 |
| *Sovia fangi* 2 | ♂ | Weixi, Yunnan Province | 20100709 | MK344897 | MK345016 | MK345144 | MK345275 | MK345406 |
| *Sovia grahami* 1 | ♂ | Cuona, Xizang Autonomous Region | 20100817 | MK344894 | — | MK345141 | MK345272 | MK345403 |
| *Sovia grahami* 2 | ♂ | Cuona, Xizang Autonomous Region | 20100818 | MK344895 | — | MK345142 | MK345273 | MK345404 |
| *Sovia lii* 1 | ♂ | Langao, Shanxi Province | 20120729 | MK344909 | — | MK345156 | MK345287 | MK345418 |
| *Sovia lucasii* 1 | ♂ | Baoxing, Sichuan Province | 201507 | MK344902 | MK345021 | MK345149 | MK345280 | MK345411 |
| *Sovia lucasii* 2 | ♀ | Baoxing, Sichuan Province | 20150721 | MK344903 | MK345022 | MK345150 | MK345281 | MK345412 |
| *Sovia lucasii* 3 | ♂ | Houhe, Hubei Province | 20130707 | MK344904 | MK345023 | MK345151 | MK345282 | MK345413 |
| *Sovia lucasii* 4 | ♂ | Maoershan, Guangxi Province | 20120723 | MK344905 | MK345024 | MK345152 | MK345283 | MK345414 |
| *Sovia lucasii* 5 | ♂ | Hanyuan, Sichuan Province | 200905 | MK344906 | MK345025 | MK345153 | MK345284 | MK345415 |
| *Sovia lucasii* 6 | ♂ | Hanyuan, Sichuan Province | 200905 | MK344907 | MK345026 | MK345154 | MK345285 | MK345416 |
| *Sovia lucasii* 7 | ♂ | Hanyuan, Sichuan Province | 200905 | MK344908 | MK345027 | MK345155 | MK345286 | MK345417 |
| *Sovia separata* 1 | ♂ | Motuo, Xizang Autonomous Region | 20130723 | MK344898 | MK345017 | MK345145 | MK345276 | MK345407 |
| *Sovia separata* 2 | ♂ | Dulongjiang, Yunnan Province | 20150716 | MK344899 | MK345018 | MK345146 | MK345277 | MK345408 |
| *Sovia separata* 3 | ♂ | Motuo, Xizang Autonomous Region | 20130727 | MK344900 | MK345019 | MK345147 | MK345278 | MK345409 |
| *Sovia separata* 4 | ♂ | Dulongjiang, Yunnan Province | 20150707 | MK344901 | MK345020 | MK345148 | MK345279 | MK345410 |
| *Sovia subflava* 1 | ♂ | Weixi, Yunnan Province | 20160720 | MK344893 | MK345014 | MK345140 | MK345271 | MK345402 |
| *Polytremis nascens* | ♂ | Qingyuan, Zhejiang | 20070721 | KJ574016* | KJ574025* | KC684355* | KC684372* | KC684338* |
| *Udaspes folus* | | | | KT240164* | KT240164* | | | |

| | | |
|---|---|---|
| *Ochlodes venata* | HM243593* | HM243593* |
| *Hylephila phyleus* | AF170859* | AF170859* |
| *Heteropterus morpheus* | KF881050* | KF881050* |
| *Daimio tethys* | KJ813807* | KJ813807* |
| *Celaenorrhinus maculosa* | KF543077* | KF543077* |
| *Achalarus lyciades* | KX249739* | KX249739* |
| *Lobocla bifasciatus* | KJ629166* | KJ629166* |
| *Choaspes benjaminii* | KJ629164* | KJ629164* |
| *Hasora anura* | KF881049* | KF881049* |
| *Potanthus flavus* | KJ629167* | KJ629167* |
| *Macrosoma sp.* | AF170854* | AF170854* |

**Table S2.** Model parameters used in ancestral area estimation analysis.

| Analysis model | Data Input | Areas | Range Constraints |
|---|---|---|---|
| S-DIVA | 15000 trees + 1 condensed tree (from BEAST) | A: Himalaya-Hengduan Mountain Region<br>B: Southern China Region<br>C: Central China Region<br>D: Northern China Region | All ranges checked in ancestral range matrix. Max areas = 4. Extinction allowed. |
| BBM | 15000 trees + 1 condensed tree (from BEAST) | A: Himalaya-Hengduan Mountain Region<br>B: Southern China Region<br>C: Central China Region<br>D: Northern China Region | All ranges checked in ancestral range matrix. Max areas = 4. The MCMC chains were run simultaneously for 5000 000 generations. The state was sampled every 100 generations. Fixed JC + G (Jukes-Cantor + Gamma) were used for analysis with null root distribution. |
